# Spontaneous Jejunal Perforation Caused by Cholesterol Crystal Embolization in a Patient with a Shaggy Aorta: A Case Report

**DOI:** 10.70352/scrj.cr.26-0107

**Published:** 2026-04-07

**Authors:** Hiromichi Tanaka, Yoshiaki Fujimoto, Kosuke Hirose, Taichi Nagano, Huanlin Wang, Fumihiko Hirai, Takuya Honboh, Noboru Harada, Seiya Kato, Noriaki Sadanaga, Tomoharu Yoshizumi

**Affiliations:** 1 Department of Surgery, Saiseikai Fukuoka General Hospital, Fukuoka, Fukuoka, Japan; 2 Department of Pathology, Saiseikai Fukuoka General Hospital, Fukuoka, Fukuoka, Japan; 3 Department of Surgery and Science, Graduate School of Medical Sciences, Kyushu University, Fukuoka, Fukuoka, Japan

**Keywords:** case report, cholesterol crystal embolization, noncontrast CT, shaggy aorta, small intestinal perforation

## Abstract

**INTRODUCTION:**

Cholesterol crystal embolization (CCE) is a systemic disease that is caused by cholesterol crystals and plaque debris that break away from atherosclerotic plaques and most commonly affects the kidneys and skin. Gastrointestinal involvement is uncommon, and perforation of the small intestine is an exceptionally rare and life-threatening complication. The preoperative diagnosis of gastrointestinal involvement is often challenging because symptoms and imaging findings are frequently nonspecific, particularly when contrast-enhanced imaging is contraindicated.

**CASE PRESENTATION:**

An 80-year-old man with end-stage renal disease (ESRD) on maintenance hemodialysis, a shaggy aorta associated with Stanford type B aortic dissection, and a history of CCE presented with mild lower abdominal pain. Initial noncontrast CT revealed localized jejunal wall thickening suggestive of enteritis or ischemia–reperfusion injury. A careful re-evaluation revealed subtle free air adjacent to the affected bowel, and a repeat CT the following day showed progression to a moderate amount of free air with intraperitoneal fluid leakage. Emergency surgical exploration revealed a focal jejunal perforation with localized ischemic change. Indocyanine green fluorescence imaging confirmed preserved macroscopic perfusion. Limited jejunal resection with primary anastomosis was performed, and a histopathological examination revealed CCE within small submucosal vessels. Therefore, the diagnosis of CCE-related intestinal ischemia was confirmed.

**CONCLUSIONS:**

Perforation of the small intestine caused by CCE is an extremely rare but critical complication that may progress rapidly despite mild initial symptoms. For patients who are at high risk, particularly those with a shaggy aorta or end-stage renal disease, careful interpretation of serial noncontrast CT findings and timely surgical intervention are essential to achieving favorable outcomes.

## Abbreviations

CCE
: cholesterol crystal embolization
ESRD
: end-stage renal disease
ICG
: indocyanine green

## INTRODUCTION

CCE is a systemic disease caused by cholesterol crystals and plaque debris that break away from atherosclerotic plaques in the aorta or its major branches and lead to small or medium artery occlusion and subsequent ischemic injury to multiple organs. The kidneys and skin are among the organs most frequently involved in CCE; however, because gastrointestinal involvement in CCE is less commonly recognized, it may be overlooked in clinical practice.^1,2)^

CCE with gastrointestinal tract involvement may present with nonspecific symptoms such as abdominal pain, diarrhea, and gastrointestinal bleeding. These symptoms often mimic other common gastrointestinal diseases, thus leading to a diagnostic delay. Previous clinical series have shown that abdominal pain, diarrhea, and gastrointestinal blood loss are the most frequent features of gastrointestinal CCE.^3)^ Because cholesterol emboli predominantly involve the microvasculature rather than major mesenteric arteries, radiological findings are often subtle and nonspecific, even on contrast-enhanced images.^4)^ Consequently, intestinal ischemia may progress to necrosis or perforation without clear early warning signs, thus creating significant diagnostic and therapeutic challenges.

Perforation of the small intestine caused by CCE is exceedingly rare; therefore, only a limited number of cases have been reported. Spontaneous onset in the absence of recent endovascular intervention is particularly uncommon. An early diagnosis of CCE with gastrointestinal involvement is complicated by ESRD because the use of contrast-enhanced CT is often contraindicated for such patients. Consistent with nationwide surgical data reported in Japan,^5)^ small intestinal perforation represents a rare but potentially fatal condition requiring prompt recognition and intervention.

We report a case of jejunal perforation caused by CCE in a patient with a shaggy aorta and ESRD. This case highlights the unexpected clinical course of CCE-related gastrointestinal injury and underscores the importance of careful interpretation of serial noncontrast images and timely surgical intervention for patients at high risk.

## CASE PRESENTATION

An 80-year-old man with a history of bilateral CCE, ESRD requiring maintenance hemodialysis, and Stanford type B aortic dissection with a patent false lumen (shaggy aorta) presented to the emergency department of our hospital with lower abdominal pain. His medical history included critical limb-threatening ischemia, hypertension, and bladder cancer after right nephrectomy. The patient was a long-term smoker; his BMI was 19.69, and he was using antiplatelet therapy, statins, ezetimibe, and low-dose corticosteroids. He had not undergone any recent endovascular interventions or invasive vascular procedures.

**Fig. 1:**
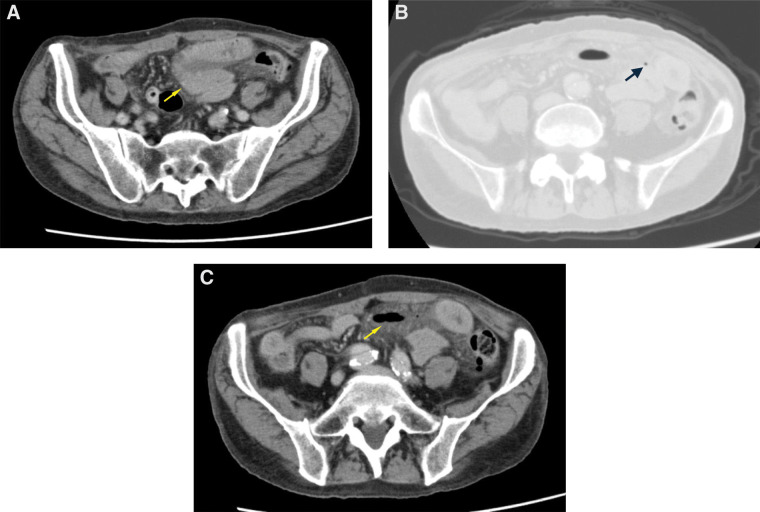
Noncontrast CT findings. Noncontrast CT image obtained at the time of initial presentation demonstrating localized wall thickening of the pelvic jejunum (arrow) (**A**) and an extremely small amount of free air adjacent to the affected small bowel (arrow) (**B**). Repeat noncontrast CT image obtained the following day, revealing a moderate amount of free air with intraperitoneal fluid leakage (arrow) (**C**).

A physical examination revealed a soft, flat abdomen with diffuse lower abdominal tenderness but without peritoneal signs on the night of admission. His vital signs were stable (blood pressure, 140/98 mmHg; heart rate, 75 bpm; temperature, 37.4°C). Laboratory tests indicated mild leukocytosis (9900/µL) and a slightly elevated C-reactive protein level (0.93 mg/dL). Noncontrast CT demonstrated localized edematous wall thickening of the pelvic small bowel with associated fat stranding and minimal ascites, suggestive of ischemic change or ischemia–reperfusion injury, rather than a definitive inflammatory process (**Fig. 1A**).

The following day, during re-evaluation of the CT images, an extremely small amount of free air adjacent to the affected small bowel was detected (**Fig. 1B**). Repeat noncontrast CT demonstrated persistent jejunal wall thickening and a moderate amount of free air with intraperitoneal fluid leakage (**Fig. 1C**). Based on these findings, microperforation of the small intestine was suspected; therefore, emergency surgical exploration was planned.

**Fig. 2:**
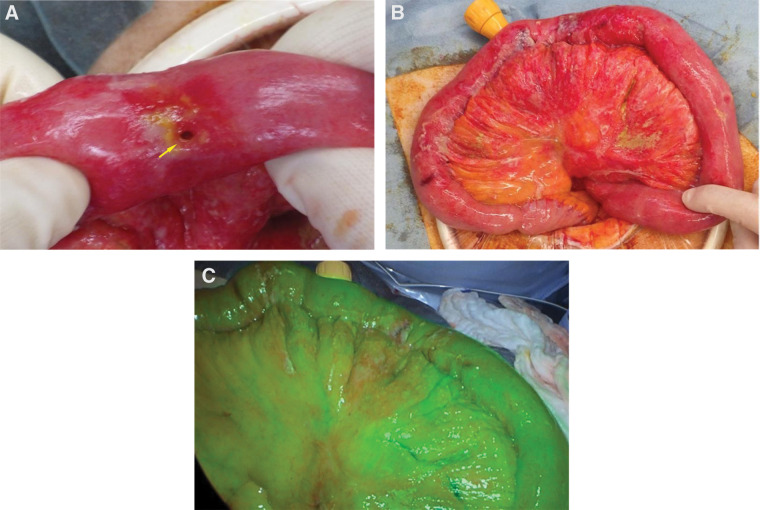
Intraoperative findings. Intraoperative findings reveal a single perforation with a diameter of approximately 5 mm in the jejunum (arrow) approximately 20 cm distal to the ligament of Treitz with localized purulent discharge (**A**). The bowel surrounding a segment of approximately 30 cm shows discoloration suggestive of ischemic change (**B**). ICG fluorescence imaging demonstrates preserved mesenteric and intestinal perfusion (**C**). ICG: indocyanine green

Emergency diagnostic laparoscopy revealed intestinal fluid leakage into the abdominal cavity, which prompted conversion to laparotomy. Intraoperatively, a single perforation with a diameter of approximately 5 mm was identified in the jejunum approximately 20 cm distal to the ligament of Treitz, and localized purulent discharge was observed (**Fig. 2A**). The bowel surrounding an approximately 30-cm segment exhibited discoloration suggestive of ischemic change (**Fig. 2B**). Indocyanine green (ICG) fluorescence imaging demonstrated preserved mesenteric and intestinal perfusion and mild intra-abdominal contamination (**Fig. 2C**). Given that the ischemic discoloration was localized around the perforated segment and that ICG fluorescence imaging confirmed preserved macroscopic perfusion in the adjacent bowel, resection was limited to the segment containing the perforation and the visibly compromised bowel. Partial jejunal resection followed by primary anastomosis and abdominal lavage with drainage were performed.

**Fig. 3:**
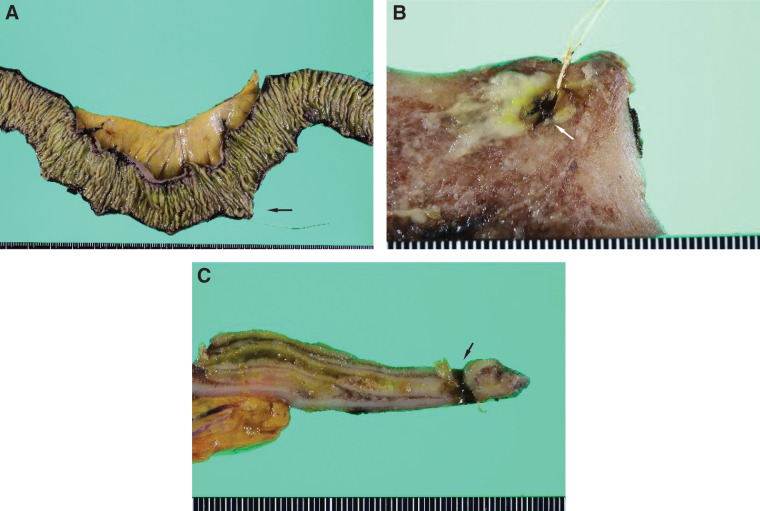
Gross appearance of the resected jejunal specimen. (**A**) Gross appearance of the formalin-fixed jejunal specimen. The perforation site is indicated by an arrow. (**B**) Close-up view of the perforated area demonstrating an approximately 5-mm defect in the jejunal wall (arrow). (**C**) Cross-sectional view of the perforation site showing the full-thickness defect of the jejunal wall (arrow).

**Fig. 4:**
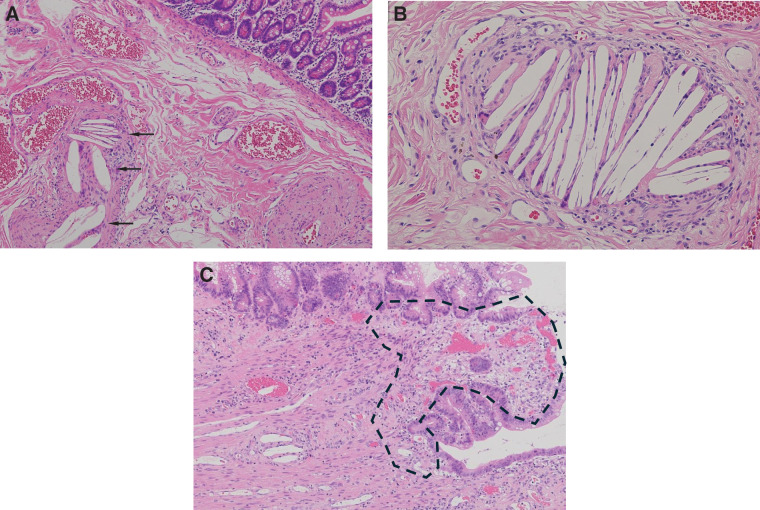
Histopathological findings. Scattered CCE within small submucosal vessels are characterized by biconvex clefts (arrows) accompanied by foreign body giant cell reactions and vascular occlusion (**A**, low-power view; **B**, high-power view) (hematoxylin and eosin staining). In the perforated area, prominent neutrophilic inflammatory infiltrates with subserosal abscess formation (dashed area) are observed in the subserosal layer (**C**), consistent with ischemic injury caused by CCE. CCE: cholesterol crystal embolization

The resected jejunal specimen demonstrated a focal perforation measuring approximately 5 mm in diameter (**Fig. 3A** and **3B**). Cross-sectional examination of the specimen confirmed a full-thickness defect of the jejunal wall at the perforation site (**Fig. 3C**). A histopathological examination revealed scattered CCE within small submucosal vessels characterized by biconvex clefts accompanied by foreign body giant cell reactions and vascular occlusion (**Fig. 4A** and **4B**). A subserosal abscess with prominent neutrophilic infiltration was observed in the perforated area (**Fig. 4C**). Eosinophilia, fibrinoid necrosis, vasculitis, and thrombus formation were not identified, thus supporting the diagnosis of intestinal ischemia caused by CCE.

Postoperatively, the patient was managed in the coronary care unit. The patient’s hemodynamic status and renal function remained stable with continued hemodialysis. No postoperative complications occurred (Clavien–Dindo grade 0). Oral intake was resumed on POD 5, and the patient was discharged home in good condition on POD 14.

## DISCUSSION

This case highlights a rare but clinically important presentation of perforation of the small intestine caused by CCE. This diagnosis is particularly challenging because CCE-induced gastrointestinal injury often progresses insidiously and lacks specific radiological and laboratory findings. This case was notable because of the occurrence of jejunal perforation in the absence of recent endovascular intervention and the role of careful reassessment of noncontrast CT findings in timely surgical intervention.

Although renal and cutaneous manifestations with CCE are well-recognized, gastrointestinal involvement is uncommon. Spontaneous gastrointestinal perforation caused by CCE has been reported only rarely, and only a limited number of cases of small intestinal perforation have been described in the literature. In this context, “spontaneous” refers to the occurrence of embolization in the absence of recent endovascular intervention or invasive vascular procedures. Patients with a shaggy aorta, patent false lumen after aortic dissection, and advanced atherosclerosis are at particularly high risk for spontaneous embolization, even without procedural triggers.^6)^ One previous case report described a spontaneous cholesterol embolism that resulted in a small bowel obstruction and perforation in the absence of recent endovascular intervention, thus highlighting the potential for severe gastrointestinal complications without an identifiable trigger.^7)^

The pathophysiology of intestinal injuries associated with CCE fundamentally differs from that of acute mesenteric ischemia. CCE causes obstruction of small submucosal arteries rather than occlusion of major mesenteric vessels, resulting in patchy and chronic ischemia.^1)^ Histopathological findings in this case demonstrated characteristic biconvex cholesterol clefts with foreign body giant cell reactions within small vessels, consistent with the findings described by prior reports.^2,8)^ These clefts represent dissolved cholesterol crystals that become embedded in the vascular lumen and trigger a foreign body inflammatory reaction, ultimately leading to microvascular occlusion and downstream ischemic injury. Such microvascular ischemia may lead to repeated cycles of injury and repair, progressive weakening of the bowel wall, and eventual localized perforation, even when macroscopic perfusion appears preserved. However, the causal relationship between CCE and intestinal perforation should be interpreted with caution. In the present case, the patient had a history of low-dose corticosteroid therapy, which has been associated with increased fragility of the intestinal wall and a potential risk of spontaneous perforation. Therefore, although histopathological findings strongly suggested CCE-related microvascular ischemia, it is possible that multiple factors, including corticosteroid use and chronic ischemic vulnerability associated with ESRD, may have contributed to the development of intestinal perforation. Nevertheless, the intraoperative findings of focal perforation with surrounding ischemic discoloration suggest localized ischemic injury potentially associated with CCE. Although ICG fluorescence imaging confirmed preserved mesenteric and intestinal perfusion, a focal jejunal perforation with surrounding ischemic discoloration was identified. These findings highlight that although ICG imaging is useful for assessing major blood flow, it cannot detect microcirculatory disturbances, which are central to the pathogenesis of CCE-related bowel injury.^9)^

The preoperative diagnosis of CCE-associated intestinal perforation is notoriously difficult. The inability of patients with ESRD to undergo contrast-enhanced CT further complicates the diagnosis. In this case, the initial noncontrast CT evaluation showed nonspecific findings suggestive of enteritis or ischemia–reperfusion injury. However, meticulous re-evaluation revealed subtle free air, and subsequent imaging demonstrated progression to a moderate amount of free air with intraperitoneal fluid leakage. This temporal change was critical because it raised the suspicion of microperforation and prompted emergency surgery. These observations underscore the importance of serial imaging and careful interpretation of minor radiological changes in patients at high risk.

When intestinal perforation occurs, surgical intervention is the only definitive treatment because no established medical therapy can reverse ongoing embolization.^1)^ The surgical strategy for CCE is challenging because of uncertainty regarding bowel viability and the potential for diffuse ischemia. Because CCE predominantly affects the microvasculature, the extent of bowel ischemia may not always correlate with macroscopic perfusion findings. Therefore, in the present case, the resection margin was determined based on a combination of macroscopic discoloration around the perforation and ICG fluorescence findings indicating preserved perfusion in the adjacent bowel. Early surgical exploration likely contributed to the favorable postoperative course.

This case highlights several clinically important aspects of CCE-related gastrointestinal injury. First, jejunal perforation occurred in the absence of recent endovascular intervention, suggesting that patients with a shaggy aorta and advanced atherosclerosis remain at risk for spontaneous cholesterol embolization. Second, the diagnosis was guided primarily by careful reassessment of serial noncontrast CT findings, which is particularly relevant for patients with ESRD in whom contrast-enhanced imaging is often avoided. Third, despite preserved macroscopic perfusion confirmed by ICG fluorescence imaging, localized intestinal perforation developed, emphasizing that microvascular ischemia caused by CCE may not be detected by conventional perfusion assessment. Together, these findings highlight the diagnostic and therapeutic challenges of CCE-related intestinal injury and underscore the importance of integrating subtle radiological changes with clinical suspicion.

Clinically, this case emphasizes that patients with known CCE, a shaggy aorta, or long-term dialysis dependence may develop life-threatening gastrointestinal complications with minimal initial symptoms. Even mild abdominal pain should prompt heightened vigilance, and noncontrast CT findings should be interpreted cautiously and longitudinally. Early surgical decision-making based on subtle but progressive imaging changes may be crucial to improving outcomes.

## CONCLUSIONS

Perforation of the small intestine caused by CCE is an exceptionally rare but life-threatening complication. This case demonstrates that CCE with gastrointestinal involvement may present with mild and nonspecific symptoms and progress rapidly, even in the absence of recent endovascular intervention. For patients who are at high risk, such as those with a shaggy aorta or ESRD, subtle and evolving noncontrast CT findings should be carefully interpreted. Early surgical decision-making based on clinical suspicion and serial noncontrast imaging, rather than reliance on macroscopic perfusion findings alone, is critical to achieving timely intervention and favorable outcomes.

## DECLARATIONS

### Availability of data and materials

The datasets supporting the conclusions of this study are included within this article.
